# Cardiovascular predictors of mortality and exacerbations in patients with COPD

**DOI:** 10.1038/s41598-022-25938-0

**Published:** 2022-12-19

**Authors:** Peter Alter, Tanja Lucke, Henrik Watz, Stefan Andreas, Kathrin Kahnert, Franziska C. Trudzinski, Tim Speicher, Sandra Söhler, Robert Bals, Benjamin Waschki, Tobias Welte, Klaus F. Rabe, Jørgen Vestbo, Emiel F. M. Wouters, Claus F. Vogelmeier, Rudolf A. Jörres

**Affiliations:** 1 Department of Medicine, Pulmonary and Critical Care Medicine, University of Marburg (UMR), Member of the German Center for Lung Research (DZL), Baldingerstrasse 1, 35033 Marburg, Germany; 2 Institute and Outpatient Clinic for Occupational, Social and Environmental Medicine, University Hospital, LMU Munich, Comprehensive Pneumology Center Munich (CPC-M), Member of the German Center for Lung Research (DZL), Munich, Germany; 3 Airway Research Center North (ARCN), Member of the German Center for Lung Research (DZL), Pulmonary Research Institute at LungenClinic Grosshansdorf, Grosshansdorf, Germany; 4 LungClinic Immenhausen and Department of Cardiology and Pneumology, University Medical Center Göttingen, Member of the German Center for Lung Research (DZL), Göttingen, Germany; 5 Department of Internal Medicine V, University Hospital, LMU Munich, Comprehensive Pneumology Center Munich (CPC-M), Member of the German Center for Lung Research (DZL), Munich, Germany; 6 Department of Pneumology and Critical Care Medicine, Thoraxklinik, University of Heidelberg, Translational Lung Research Center Heidelberg (TLRC-H), Member of the German Center for Lung Research (DZL), Heidelberg, Germany; 7 Department of Internal Medicine V - Pulmonology, Allergology, Intensive Care Medicine, Saarland University Hospital, Homburg, Germany; 8 Department of Pneumology, Hospital Itzehoe, Airway Research Center North (ARCN), Member of the German Center for Lung Research (DZL), LungenClinic Grosshansdorf, Grosshansdorf, Germany; 9 Department of Cardiology, University Heart Center Hamburg, Hamburg, Germany; 10 Clinic for Pneumology, Hannover Medical School, Member of the German Center for Lung Research (DZL), Hannover, Germany; 11 LungenClinic Grosshansdorf and Department of Medicine, Airway Research Center North (ARCN), Member of the German Center for Lung Research (DZL), Christian-Albrechts University, Kiel, Kiel/Grosshansdorf, Germany; 12 Division of Infection, Immunity and Respiratory Medicine, University of Manchester, Manchester, UK; 13 Department of Respiratory Medicine, Maastricht University Medical Centre, Maastricht, The Netherlands; 14 Ludwig Boltzmann Institute for Lung Health, Vienna, Austria

**Keywords:** Clinical trials, Cardiovascular diseases, Respiratory tract diseases

## Abstract

In chronic obstructive pulmonary disease (COPD), comorbidities and worse functional status predict worse outcomes, but how these predictors compare with regard to different outcomes is not well studied. We thus compared the role of cardiovascular comorbidities for mortality and exacerbations. Data from baseline and up to four follow-up visits of the COSYCONET cohort were used. Cox or Poisson regression was employed to determine the relationship of predictors to mortality or mean annual exacerbation rate, respectively. Predictors comprised major comorbidities (including cardiovascular disease), lung function (forced expiratory volume in 1 s [FEV_1_], diffusion capacity for carbon monoxide [TLCO]) and their changes over time, baseline symptoms, exacerbations, physical activity, and cardiovascular medication. Overall, 1817 patients were included. Chronic coronary artery disease (*p* = 0.005), hypertension (*p* = 0.044) and the annual decline in TLCO (*p* = 0.001), but not FEV_1_ decline, were predictors of mortality. In contrast, the annual decline of FEV_1_ (*p* = 0.019) but not that of TLCO or cardiovascular comorbidities were linked to annual exacerbation rate. In conclusion, the presence of chronic coronary artery disease and hypertension were predictors of increased mortality in COPD, but not of increased exacerbation risk. This emphasizes the need for broad diagnostic workup in COPD, including the assessment of cardiovascular comorbidity.

**Clinical Trials:** NCT01245933.

## Introduction

Comorbidities are known to have a significant impact on prognosis in patients with chronic obstructive pulmonary disease (COPD)^1–5^, and their burden is associated with worse lung function^6^. Among the frequent comorbidities are cardiovascular diseases, such as coronary artery disease and myocardial infarction^7,8^. Although COPD exacerbations are known to be linked to mortality risk, both could depend in a different manner on comorbidities, particularly those of the cardiovascular spectrum. To answer this question requires a comparative analysis of their role for both outcomes.

It could also be relevant that patients with comorbid COPD and cardiovascular disease usually receive treatment for both entities. COPD therapy ameliorates the rate of lung function decline^9^, while cardiovascular medication has beneficial effects on mortality from cardiovascular disease^10,11^. It is, however, not clear, to which extent this medication also impacts mortality in patients, in whom the lung disease dominates the clinical state. As exacerbations are a risk factor for mortality and one of the major targets of respiratory medication^12^ the potential effect of cardiovascular medication on the rate of COPD exacerbations is of interest^13,14^. Until now, only few systematic analyses have addressed the role of cardiovascular disease^15^ and medication for both mortality and exacerbations of COPD patients.

Lung function decline is known to be an unfavourable prognostic factor in COPD. It shows marked heterogeneity between patients^16^, and a rapid decline in forced expiratory volume in 1 s (FEV_1_), for example, is associated with increased mortality from coronary heart disease^17^. The decline of FEV_1_ is assumed to be predominantly related to airway pathology, while that of carbon monoxide (CO) diffusing capacity is associated with lung emphysema^18^. This suggests that a comparative analysis of risk factors for mortality and exacerbations should include the rate of decline of several lung function measures, to accommodate COPD phenotypes.

Based on this, the aim of this study was to compare the role of a broad spectrum of predictors for both mortality and exacerbations in patients with COPD. The predictors included multiple comorbidities, cardiovascular medication, lung function, its decline, and physical activity. For this purpose we analyzed data from a large observational COPD cohort.

## Methods

### Study population and assessments

**Figure 1:**
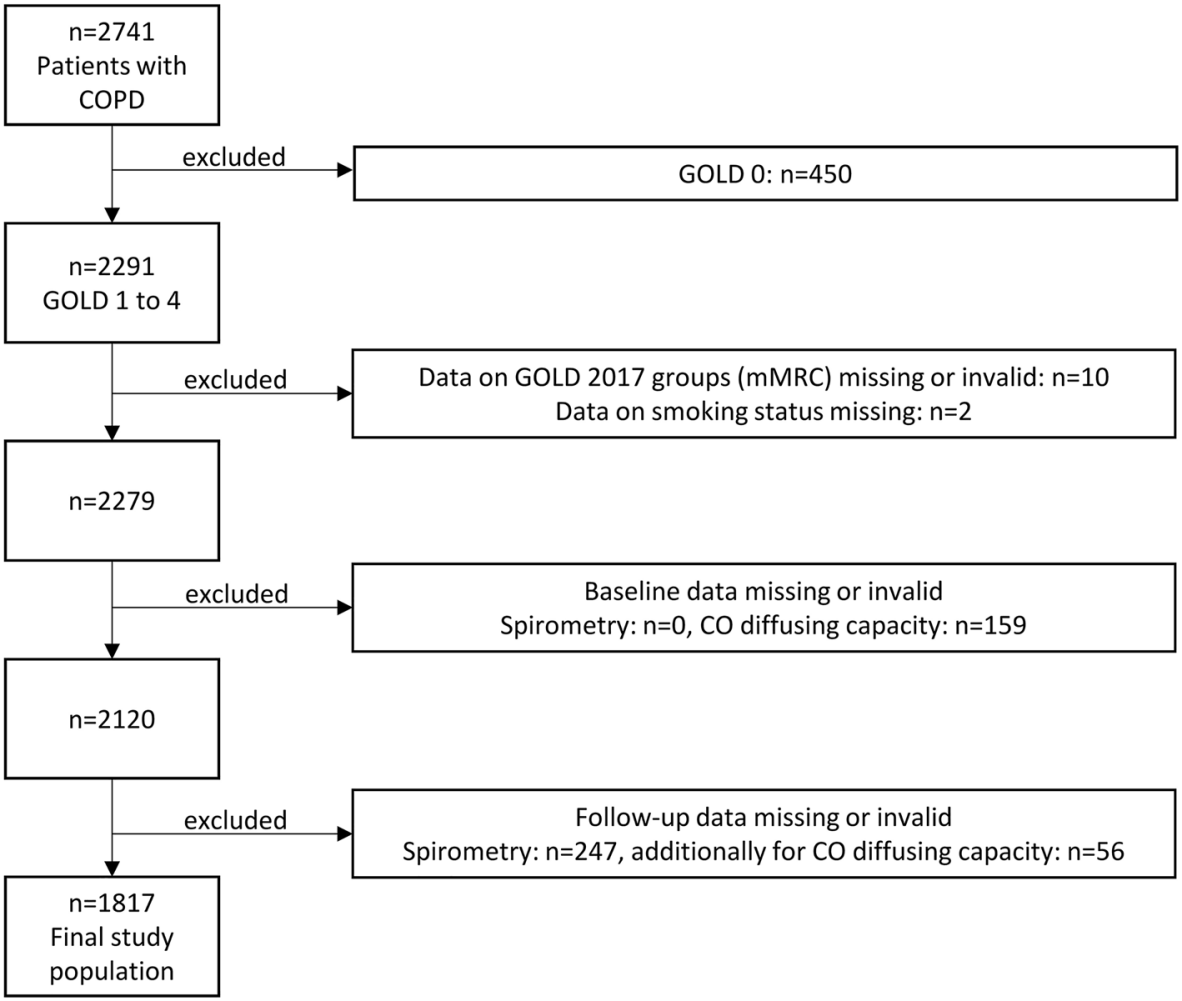
Study flow chart showing the selection process of patients. GOLD, Global Initiative for Chronic Obstructive Lung Disease.

COSYCONET (COPD and Systemic Consequences—Comorbidities Network) is a multi-centre, long-term observational cohort study in patients with stable COPD. Patients were enrolled in 31 recruiting centres spread across Germany, and patients were required to be free of exacerbations during 4 weeks prior to enrolment. Details of the study design are available elsewhere^19^. Coronary artery disease, a history of myocardial infarction, heart failure, and various non-cardiovascular comorbidities were assessed on entry to the study in a structured interview comprising patients’ reports of physician-based diagnoses^3^. To account for potential different meanings, chronic coronary artery disease not defined by a prior myocardial infarction was analysed separately. Regular follow-up visits 2–5 were performed 6, 18, 36 and 54 months after enrolment (visit 1), with comprehensive lung function testing, recording of clinical characteristics and medication at each visit. The present analysis included patients with FEV_1_ to forced vital capacity (FVC) ratio < 0.7 on entry (Global Initiative for Chronic Obstructive Lung Disease [GOLD] grades 1–4) in whom changes in lung function (calculated as annual change) between at least two visits could be determined (see Flow chart, Fig. 1).

Lung function was assessed post-bronchodilator, with FEV_1_ and FVC used as measures of airway mechanics, and single-breath diffusing capacity for CO as measure of gas exchange and alveolar integrity, in terms of the transfer factor (TLCO) and transfer coefficient (KCO, ratio of TLCO to alveolar volume). At baseline, patients were categorized by airflow limitation (GOLD grades 1–4) and allocation to GOLD 2017 ABCD groups as recommended, indicating exacerbation risk and symptoms^20,21^. Increased exacerbations were defined when at least 2 moderate or 1 severe exacerbations occurred during the previous 12 months. For symptom assessment, we used the mMRC (modified Medical Research Council) dyspnoea scale^22^, with exacerbation risk assessment based on patient-reported exacerbations in the previous year. GOLD groups B and D were pooled to indicate increased symptoms, and groups C and D to indicate increased exacerbation risk; the pooled CD category is essentially equivalent to the most recently introduced category GOLD E^23^. For the assessment of physical activity, the International Physical Activity Questionnaire (IPAQ) was used.

Echocardiography was employed to determine left ventricular end-diastolic and end-systolic diameters (LVEDD and LVESD), left ventricular ejection fraction (LVEF), and left atrial diameter (LA), with the same quality criteria as applied in previous COSYCONET analyses^3,24,25^. Baseline measures were used for functional characterisation and to assess the proportion of patients with cardiac impairment^10^.

### Cardiovascular and respiratory medication

The use of cardiovascular medication was assessed at each visit via Anatomical Therapeutic Chemical classification codes, focusing on substances used in cardiac disease, grouped into the following categories: beta-blockers; substances interacting with the renin-angiotensin system including angiotensin converting enzyme inhibitors, AT_1_ receptor blockers, direct renin inhibitor and mineralocorticoid receptor antagonists, collectively called RAAS inhibitors; acetylsalicylic acid; lipid-lowering drugs including statins, fibrates and ezetimibe. For statistical analysis, we identified groups of patients maintaining cardiovascular medication of each type at their last two study visits. These groups were compared with the respective complementary groups, i.e., patients in whom the respective medication was changed or absent. Inhaled maintenance respiratory medication was also recorded.

### Outcome assessment

The follow-up procedures have been described previously^26^. Visit 5 was scheduled 54 months after study enrolment. The first outcome was all-cause mortality, for which all available data were used. For patients participating in the study beyond visit 5, a follow-up survey between visit 5 and the scheduled visit 6 was employed. The time of survival or loss to follow-up was calculated as time interval from study enrolment to the latest contact or known death. The second outcome was the mean annual rate of exacerbations determined until each patient’s last visit. For descriptive purposes, patients were grouped into either survivors versus deceased patients, or into those having ≥ 2 exacerbations per year on average versus those having ≤ 1 exacerbation per year on average, with exacerbations again defined following GOLD 2017 recommendations^20^.

### Data analysis

For descriptive analyses, mean values and standard deviations (SD) were used. When analysing the changes in lung function over time, extreme changes exceeding 40% predicted/year were excluded. These data, as well as baseline data, were used for comparisons between survivors and deceased participants, as well as between patients in the high and low exacerbation occurrence categories. To account for collinearities, multivariate Cox regression analysis was employed by successively adding sets of related predictors, starting with age, sex, and body-mass index (BMI), adding lung function and rates of change, non-cardiovascular comorbidities, cardiovascular comorbidities, symptoms and exacerbation risk at baseline, physical activity, and finally cardiovascular medication. It should be noted that the successively added sets of predictors were predetermined and not selected on a statistical basis, and the procedure was chosen to check for the robustness against confounding. In the present work, only the final results are presented due to space considerations. Predictors of exacerbation rate, in terms of the average annual number of exacerbations, were determined using multivariate Poisson regression analysis using the same set of predictors as for mortality. *P* values < 0.05 were considered statistically significant (two-sided). All analyses were performed using IBM SPSS Statistics (Version 27.0.0.0, Armonk, NY, US).

### Ethics approval and consent to participate

COSYCONET was approved by the ethical committees of all study centres and all patients gave written informed consent. It was performed in accordance with the declaration of Helsinki. Clinicaltrials.gov: NCT01245933. The study protocol was approved by the central ethical committee in Marburg (Ethikkommission FB Medizin Marburg) and the respective local ethical committees: Bad Reichenhall (Ethikkommission Bayerische Landesärztekammer); Berlin (Ethikkommission Ärztekammer Berlin); Bochum (Ethikkommission Medizinische Fakultät der RUB); Borstel (Ethikkommission Universität Lübeck); Coswig (Ethikkommission TU Dresden); Donaustauf (Ethikkommission Universitätsklinikum Regensburg); Essen (Ethikkommission Medizinische Fakultät Duisburg-Essen); Gießen (Ethikkommission Fachbereich Medizin); Greifswald (Ethikkommission Universitätsmedizin Greifswald); Großhansdorf (Ethikkommission Ärztekammer Schleswig-Holstein); Hamburg (Ethikkommission Ärztekammer Hamburg); MHH Hannover/Coppenbrügge (MHH Ethikkommission); Heidelberg Thorax/Uniklinik (Ethikkommission Universität Heidelberg); Homburg (Ethikkommission Saarbrücken); Immenhausen (Ethikkommission Landesärztekammer Hessen); Kiel (Ethikkommission Christian-Albrechts-Universität zu Kiel); Leipzig (Ethikkommission Universität Leipzig); Löwenstein (Ethikkommission Landesärztekammer Baden-Württemberg); Mainz (Ethikkommission Landesärztekammer Rheinland-Pfalz); München LMU/Gauting (Ethikkommission Klinikum Universität München); Nürnberg (Ethikkommission Friedrich-Alexander-Universität Erlangen Nürnberg); Rostock (Ethikkommission Universität Rostock); Berchtesgadener Land (Ethikkommission Land Salzburg); Schmallenberg (Ethikkommission Ärztekammer Westfalen-Lippe); Solingen (Ethikkommission Universität Witten-Herdecke); Ulm (Ethikkommission Universität Ulm); Würzburg (Ethikkommission Universität Würzburg).

## Results

### Baseline description

Overall, 2741 patients with stable COPD were enrolled in COSYCONET^19^. A subset of 2291 patients were GOLD grades 1–4, 2120 of whom had valid data for FEV_1_, FVC, TLCO and KCO, and could have GOLD A-D grouping determined at visit 1 (Fig. 1). Changes in lung function between first and last visit could be evaluated in 1817 patients, which is the final study population. Baseline characteristics are given in Table 1. The visit 1 data for these 1817 patients are shown in Table 1. Mean (± SD) values of FEV_1_ and TLCO were 54.3 ± 18.1% and 56.9 ± 21.2% predicted, respectively.

**Table 1:** Characteristics of the study population upon inclusion (visit 1).

Baseline characteristics	Study population n = 1817
Sex (m/f) (n, %)	1118 (61.5%) / 699 (38.5%)
Age (y)	64.8 ± 8.3
BMI (kg/m^2^)	26.8 ± 5.2
Smoking status (current) (n, %)	441 (24.3%)
Physical activity (IPAQ)	4489.1 ± 4856.6
**Lung function**	
FEV_1_ (% predicted GLI)	54.3 ± 18.1
FVC (% predicted GLI)	79.9 ± 18.5
TLCO (% predicted GLI)	56.9 ± 21.2
KCO (% predicted GLI)	65 ± 22.1
**COPD severity, symptoms and exacerbation risk**	
mMRC (score)	1.54 ± 0.89
GOLD 1/2/3/4	181/811/681/144
GOLD A/B/C/D (mMRC based)	755/446/243/373
Increased symptoms at baseline	819 (45.1%)
High exacerbation risk at baseline	616 (33.9%)
Average number of exacerbations per year	1.1 ± 1.0
Physical activity (IPAQ)	4489 ± 4857
6-MWD (m)	425 ± 102
**Respiratory medication**	
Any LABA	1515 (83.4%)
Any LAMA	1348 (74.2%)
Any ICS	1173 (64.6%)
Triple therapy	918 (50.5%)

Data are given as mean values ± standard deviation or numbers (percentages). FEV_1_ = forced expiratory volume in 1 s; FVC = functional vital capacity; TLCO = diffusing capacity for carbon monoxide (transfer factor); KCO = CO transfer coefficient; mMRC = modified Medical Research Council; IPAQ = International Physical Activity Questionnaire; LABA = long-acting beta2-agonists; LAMA = long-acting muscarinic antagonists; ICS = inhaled corticosteroids. Increased COPD symptoms refer to allocation to GOLD groups B or D and high exacerbation risk to GOLD groups C or D.

Of the 1817 patients, 217, 257, 307 and 836 had their last examination at visits 2, 3, 4 or 5, respectively. The mean (± SD) declines in FEV_1_ and TLCO per year (expressed as change in baseline percent predicted) were 1.48 ± 5.46% and 2.58 ± 7.56%, respectively (Table 2). Their most frequent non-cardiovascular and cardiovascular comorbidities are shown in Table 3.

**Table 2:** Changes of lung function during follow-up.

Characteristics during follow-up	Study population n = 1817
Changes of lung function from baseline per year	
**Expressed as percent baseline**	
Delta/y FEV_1_ (% Baseline)	−2.61 ± 12.04
Delta/y FVC (% Baseline)	−1.84 ± 11.90
Delta/y TLCO (% Baseline)	−3.52 ± 23.37
Delta/y KCO (% Baseline)	−2.31 ± 21.29
**Expressed as percent predicted at baseline**	
Delta/y FEV_1_ (% predicted GLI)	−1.48 ± 5.46
Delta/y FVC (% predicted GLI)	−1.77 ± 8.01
Delta/y TLCO (% predicted GLI)	−2.58 ± 7.56
Delta/y KCO (% predicted GLI)	−2.17 ± 8.03
**Time of observation**	
Observation interval days (25%, 50%, 75% quartiles)	1071/1583/1659

The changes in lung function (delta/y) are expressed as a raw percent changes per year relative to baseline, moreover as changes per year relative to baseline in terms of percent predicted at baseline in order to reduce variation. Only the latter were used for analysis. FEV_1_ = forced expiratory volume in 1 s; FVC = functional vital capacity; TLCO = diffusing capacity for carbon monoxide; KCO = CO transfer coefficient.

**Table 3:** Comorbidities of the total study population and echocardiographic measurements at visit 1.

Comorbidities	n (%)
Coronary artery disease	330 (18.2%)
Coronary artery disease w/o infarction	172 (9.5%)
History of myocardial infarction	158 (8.7%)
Heart failure	88 (4.8%)
Cardiac rhythm disorders	156 (8.6%)
Arterial hypertension	1020 (56.1%)
Asthma	334 (18.4%)
Sleep apnoea	201 (11.1%)
Diabetes	236 (13.0%)
Hyperlipidaemia	804 (44.2%)
Hyperuricaemia	334 (18.4%)
Osteoporosis	281 (15.5%)
Psychological disorders	437 (24.1%)
**Echocardiographic measurements (n = 1347)**	
LVEDD (mm)	48.11 ± 7.01
LVESD (mm)	32.21 ± 7.15
LVEF (%)	61.59 ± 9.07
LA (mm)	36.03 ± 6.37

The echocardiographic measures are given as mean values and standard deviations; the number of patients with impairments is given in the Results. LVEDD = left ventricular end-diastolic diameter; LVESD = left ventricular end-systolic diameter; LVEF = left ventricular ejection fraction, LA = left atrial diameter.

The mean (± SD) echocardiographic LVEF at baseline was 61.6 ± 9.1% (Table 3), with few patients having severely reduced LVEF: 29 patients (1.6%) had values < 40%, 10 (0.7%) < 35%, and 5 (0.4%) < 30%. Cardiovascular medication including diuretics was taken by more than half of the study participants at both of the last two study visits, most commonly RAAS inhibitors (Table 4).

**Table 4:** Numbers of patients with constant cardiovascular medication at the last two study visits.

Cardiovascular medication at the last two visits of participation	n (%)
Antiplatelet agents	450 (24.8%)
Beta-blockers	411 (22.6%)
Lipid-lowering drugs	435 (23.9%)
RAAS inhibitors	786 (43.3%)
Diuretics	531 (29.2%)

RAAS = Renin–angiotensin–aldosterone system. For details see Methods. Percentages refer to the total study population (n = 1817).

### Predictors of mortality

The median (quartiles) time of observation until patients’ last contact or known death was 1583 (1071; 1659) days, with a maximum of 2674 days. Over this period, 153 patients died (8.4% of the study population; 115 [75.2%] males, 38 [24.8%] females). Deceased patients tended to be older, had worse lung function at baseline, a greater annual decline of FEV_1_ and TLCO, higher exacerbation risk and increased COPD symptoms at baseline, and exhibited comorbid conditions more frequently, in particular coronary artery disease without a history of myocardial infarction, and hypertension (Additional File 1, Supplementary Table 1). To illustrate their role for mortality in univariate analyses, Kaplan–Meier curves are shown in Additional File 1, Supplementary Figs. 1A,B.

Cox regression analyses were performed to identify potential independent predictors of mortality, including the following variables: sex, age, BMI, smoking status, FEV_1_ and TLCO % predicted at baseline and their changes over time (as % predicted), presence of coronary artery disease without myocardial infarction, heart failure, rhythm disorders, hypertension, asthma, sleep apnoea, diabetes, hyperlipidaemia, hyperuricaemia, osteoporosis, psychological disorders, IPAQ, high exacerbation risk and increased symptoms at baseline (GOLD CD and BD groups, respectively), and cardiovascular medication (antiplatelet agents, beta-blockers, RAAS inhibitors, diuretics, and lipid-lowering drugs).

**Figure 2:**
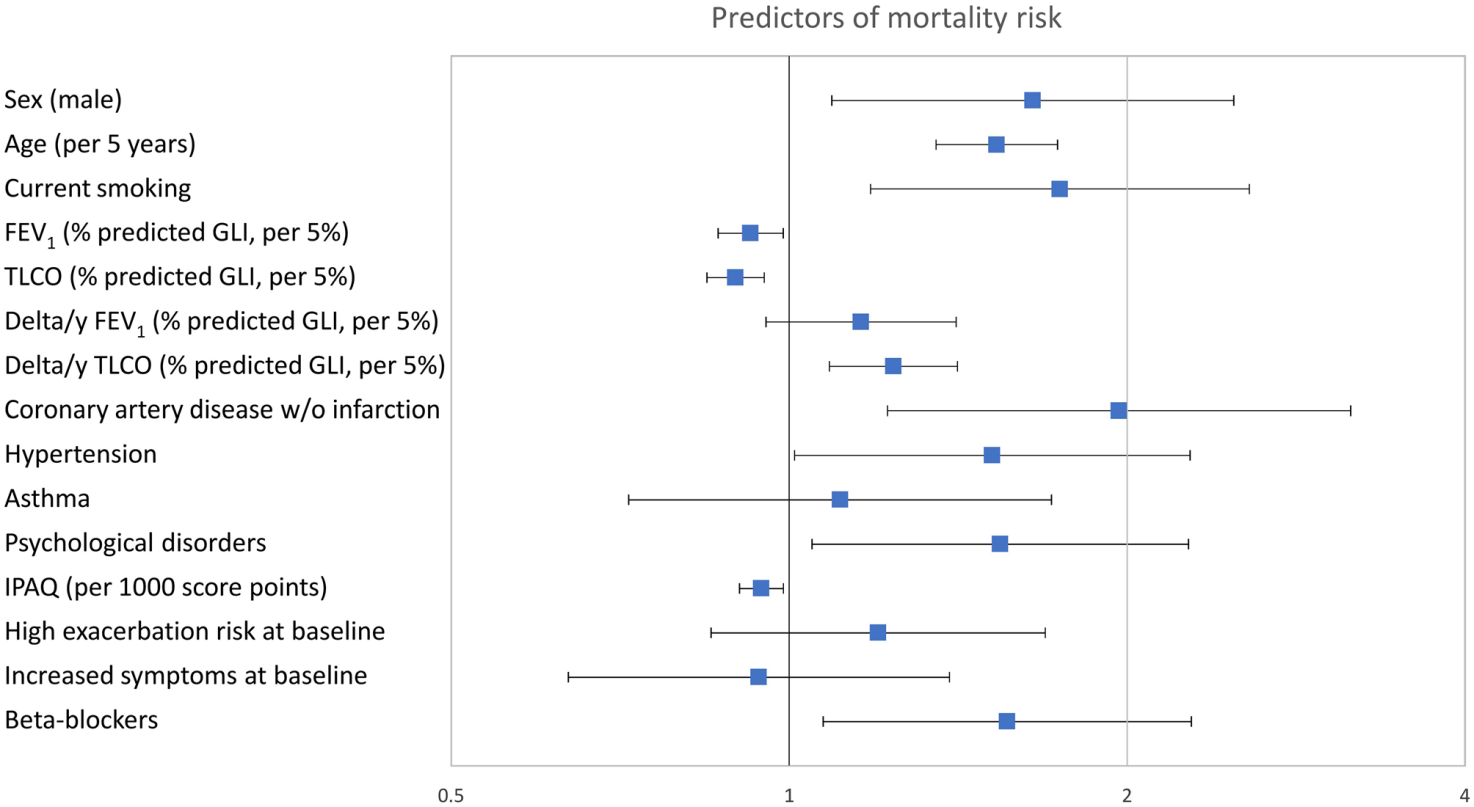
Predictors of mortality risk. Hazard ratios and 95% confidence intervals for predictors of mortality according to Cox regression analysis. The numerical values correspond to those of Supplementary Table 2. The predictors shown are those that were statistically significant (*p* < 0.05) in multivariable analyses of either increased mortality or increased occurrence of exacerbations to ensure comparability with Fig. 3, additionally asthma as important respiratory comorbidity. All predictors except for the decline in lung function (delta/y) refer to baseline values at the initial visit. Increased COPD symptoms correspond to GOLD groups B or D and high exacerbation risk to GOLD groups C or D at baseline. FEV_1_ = forced expiratory volume in 1 s; TLCO = diffusing capacity for carbon monoxide; IPAQ = International Physical Activity Questionnaire.

Significant predictors of mortality were: male sex, increased age, current smoking, lower baseline FEV_1_ and TLCO % predicted, increased annual decline in TLCO % predicted, self-reported, physician-based history of coronary artery disease without myocardial infarction, hypertension, psychological disorders, lower IPAQ and the use of beta-blockers at the last two visits (all *p* < 0.05; Fig. 2 and Additional File 1, Supplementary Table 2). Further details and sensitivity analyses are in Additional File 1. When replacing the baseline exacerbation category CD by the mean annual number of exacerbations over the individual study period, exacerbation rate became an additional predictor of mortality (*p* < 0.001) but the differential association with TLCO and its decline versus that of FEV_1_, as well as with CAD w/o infarction remained significant, while that for hypertension showed a p-value of 0.064 instead of 0.044. The result remained the same, when replacing the mMRC-based baseline symptom category GOLD BD by either the presence of BD at any of the follow-up visits or the mean value of mMRC, however in this case the association of symptoms with mortality additionally became statistically significant (*p* < 0.05 each).

### Predictors of annual exacerbation rate

Similar to the mortality analyses, baseline characteristics, lung function, comorbidities, and medication were compared between patients in the low vs high exacerbation categories according to their average annual rate; 578 patients (31.8%) were in the high exacerbation category. Patients in this category had worse lung function at baseline, higher annual decline in TLCO, lower activity according to IPAQ and more comorbidities (Additional File 1, Supplementary Table 3).

**Figure 3:**
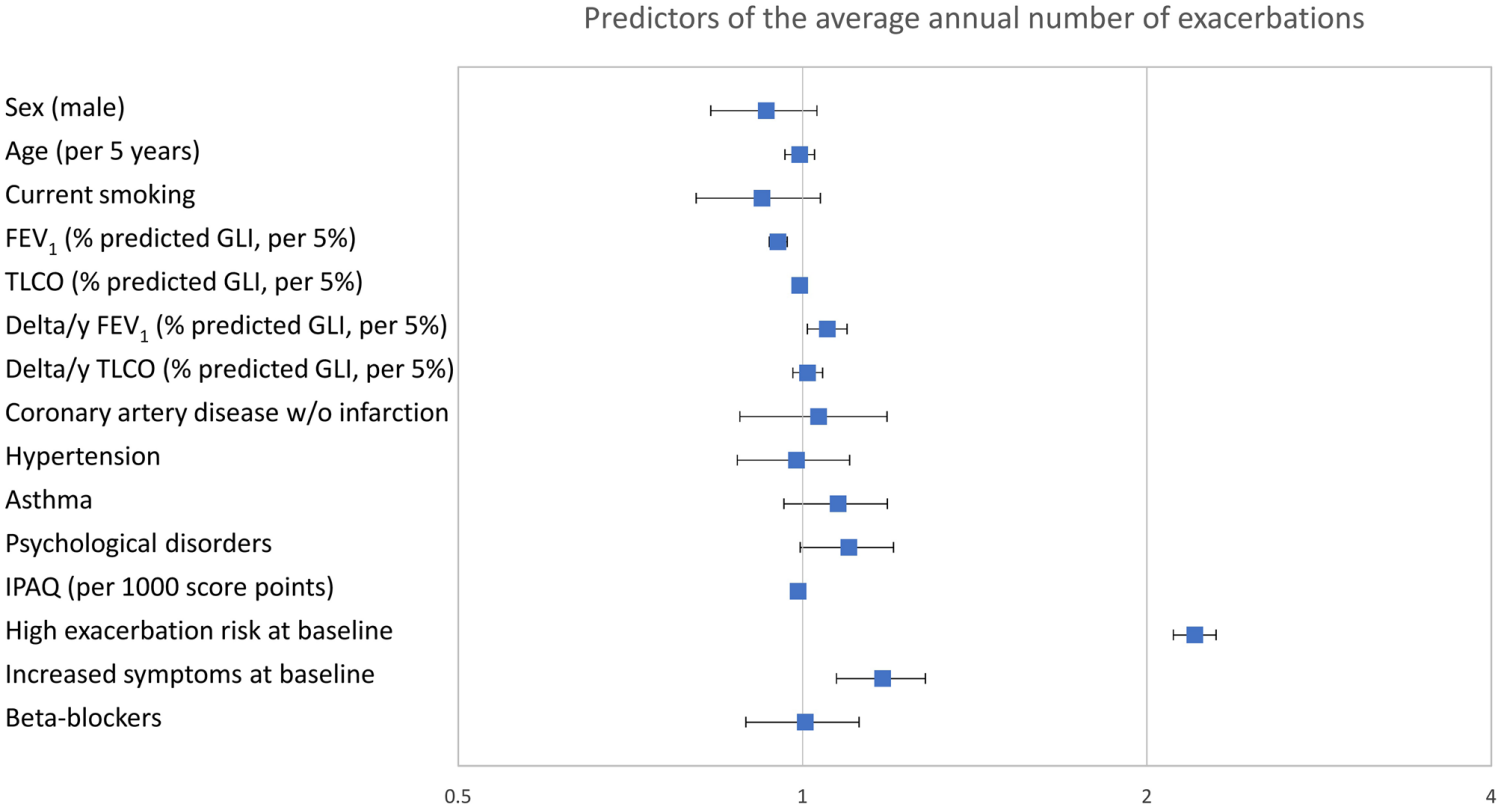
Predictors of the average annual number of exacerbations. Odds ratios and 95% confidence intervals for predictors of the mean annual number of exacerbations according to Poisson regression analysis. The numerical values correspond to those of Supplementary Table 4. The predictors shown are those which were statistically significant (*p* < 0.05) in multivariable analyses of either increased mortality or rate of exacerbations to ensure comparability with Fig. 2, additionally asthma as important respiratory comorbidity. All predictors except for the decline in lung function (delta/y) refer to baseline values at the initial visit. Increased COPD symptoms correspond to GOLD groups B or D and high exacerbation risk to GOLD groups C or D at baseline. FEV_1_ = forced expiratory volume in 1 s; TLCO = diffusing capacity for carbon monoxide; IPAQ = International Physical Activity Questionnaire.

To identify independent predictors of the annual number of exacerbations, Poisson regression analysis was employed with the same set of predictors as used in the mortality analysis. Using this multivariable approach, baseline FEV_1_ and its annual decline, as well as baseline exacerbation risk and symptoms were identified as independent predictors of exacerbation rate (*p* < 0.05 each; Fig. 3, and Additional File 1, Supplementary Table 4 and supplemental results). The parameter with the highest odds ratio (i.e., the strongest predictor) was baseline exacerbation risk. As average exacerbation rate was the outcome, an additional analysis involving the replacement of baseline exacerbation rate with average rate could not be performed. The association of the mean annual exacerbation rate with symptoms remained after replacing the mMRC-based baseline symptom category GOLD BD by either the presence of BD at any of the follow-up visits or the mean value of mMRC (*p* ≤ 0.001 each).

## Discussion

Using data from an observational COPD cohort, we found that coronary artery disease and hypertension were associated with increased all-cause mortality independent of other predictors, in line with previous data. Remarkably, the association with coronary artery disease was found only for patients who did not report a myocardial infarction as indicating event. These patients probably represent a specific subset of patients with chronic cardiovascular conditions, underlining the value of a detailed categorization of these disorders. Moreover, CO diffusing capacity at baseline and its decline over time were strong predictors of mortality, while FEV_1_ and its decline were of minor or no importance. Additional predictors of mortality were a low level of daily activity and the use of beta-blockers, as well as average but not baseline exacerbation rate. In contrast, average annual exacerbation rate was associated with the annual decline of FEV_1_ but not that of CO diffusing capacity, and with none of the cardiovascular comorbidities. Thus, parameters of lung function contributed differently to the prediction of mortality and exacerbation rate, potentially in relation to different COPD and comorbidity phenotypes and consistent with previous data^27–29^. Covering a broad spectrum of predictors including cardiovascular medication, the detailed comparison of associations with mortality and exacerbation rate specifically revealed the different role of comorbidities and functional parameters.

In common with other COPD cohorts^1,8^, cardiovascular comorbidities were frequent in our study population^3,5,19^, and functional and clinical baseline characteristics were typical of large COPD studies. Thus, the cohort appeared appropriate for examining the extent to which cardiovascular comorbidities in COPD are associated with worse prognosis. Large community- or population-based studies have already provided evidence for this association, although these studies either did not specifically target patients with COPD, or collected more limited data on lung function decline^17,30,31^; this is important as comorbidities are associated with increased lung function decline in general^6,32^. In a population of primary care COPD patients, an accelerated FEV_1_ decline was not linked to increased morbidity or mortality from cardiovascular disease, in line with our findings^28^; this also applied to average exacerbation rate as predictor of mortality if this was introduced in our analysis instead of its baseline category. The prognostic impact of cardiovascular disease in COPD has also been addressed in subgroup analyses of interventional trials on respiratory medication with follow-up periods of 1–3 years^2,33^. Our observational design had a comparable follow-up period (lower quartile 2.9 years), but might have benefited from the presence of medication prior to inclusion, distinct from interventional trials in which medication was newly introduced at baseline.

Coronary artery disease was present in 18.2% of patients, and 8.7% reported a prior myocardial infarction. Correspondingly, chronic coronary disease not indicated by myocardial infarction occurred in 9.5% of patients, similar to data from other COPD cohorts^1,16^. Among cardiac disorders, only chronic coronary artery disease was linked to mortality, while previous infarction per se was not. This may suggest that a chronic character of cardiovascular conditions is of particular importance in COPD, while a non-fatal and locally limited event such as myocardial infarction does not necessarily result in a long-standing impairment. It is noteworthy that in our cohort the mean LVEF was 62%, and the proportion of patients with at least moderately reduced LV function was below 2%. This was probably why the potential contribution to mortality from myocardial impairment was negligible, while that from coronary artery disease without a prior diagnosis of myocardial infarction was detectable. Hypertension was the most common cardiovascular disorder, occurring in more than half of the study population, in accordance with previous data^1^, but showed only a weak association with mortality.

The fact that we studied a cohort of patients with COPD in whom cardiac disease was a comorbidity, and not a primary cardiologic cohort, could explain why we did not observe significant associations between mortality and cardiovascular medication, despite the inclusion of the full panel of antiplatelet drugs, RAAS inhibitors, lipid-lowering drugs and diuretics. The situation was different for beta-blockers, which were associated with increased mortality. The most likely explanation is that their use reflected the severity of cardiac conditions for which we did not have sensitive independent markers for adjustment (see Additional File 1, supplemental discussion).

In addition to cardiovascular predictors, male sex, increased age and current smoking were relevant for mortality, consistent with known data^34–36^. Moreover, baseline TLCO, and to a lesser extent baseline FEV_1_, were relevant, with impairments being linked to increased mortality^37^. It is interesting that only TLCO decline was associated with mortality, while the rate of exacerbations was linked only to the decline in FEV_1_. This emphasized the importance of a comprehensive lung function assessment including diffusing capacity as COPD assessment tool.

Since we did not have computed tomography images in a sufficient number of patients, we could not analyse the relationships to emphysema determined by imaging. It is known, however, that CO diffusing capacity is closely related to emphysema, more so than other lung function measures including FEV_1_^38^, and thus is the best functional marker of emphysema currently available. Our TLCO findings are consistent with data showing emphysema as a risk factor for mortality^37^. One explanation could be that the long-term loss of alveolar integrity has multiple systemic consequences, for example through impairment of gas exchange^39^ or effects on the heart via lung hyperinflation^24^. It is less plausible that reductions in diffusing capacity contribute to acute exacerbations to the same degree. In contrast, short-term variations in lung function linked to airway inflammation and exacerbations might be better reflected by measures of airway obstruction. Furthermore, the finding that exacerbation history at baseline was the strongest predictor of the subsequent mean rate of exacerbations is consistent with data underlying the GOLD grouping^20^, while increased symptoms at baseline were also a significant predictor of exacerbation rate in our analyses.

Although the study population was large, the proportion with cardiac comorbidities was relatively small (20.5%), thus limiting the power to differentiate between cardiac diseases. In addition, the presence of cardiac disease relied on patient reports and no data on coronary angiography were available. Due to the observational design, we do not imply causality from the findings. Nevertheless, coronary artery disease without a history of infarction was clearly identified as risk factor for increased mortality (see Additional File 1, supplemental discussion).

## Conclusions

Chronic coronary artery disease was a relevant contributor to mortality in COPD. While changes in lung diffusing capacity but not in airflow limitation over time were relevant for mortality, those in airflow limitation but not in diffusing capacity were related to the mean annual rate of exacerbations. Exacerbations were not associated with cardiac diseases. Thus, the panels of predictors of mortality and mean exacerbation rate in patients with COPD were different, despite the clinical link between both outcomes, and the presence of cardiovascular comorbidity appeared to increase mortality risk without being indicated by exacerbation rate. Our findings demonstrate the gain in clinical predictive ability in COPD through a broad diagnostic workup comprising multiple lung function parameters and a detailed assessment of cardiovascular disease.

## Supplementary Information

Supplementary Information.

## Data Availability

The basic data are part of the German COPD cohort COSYCONET (www.asconet.net) and available upon request. The website of the network provides a detailed procedure for respective applications. The data can be obtained after submission of a proposal that is evaluated by the steering committee. All results to which the manuscript refers are documented appropriately in the text, figures or tables.

## Abbreviations

BMI: Body-mass index
CO: Carbon monoxide
COPD: Chronic obstructive pulmonary disease
COSYCONET: COPD and systemic consequences—comorbidities network
FEV_1_: Forced expiratory volume in 1 s
FVC: Forced vital capacity
GOLD: Global Initiative for chronic obstructive lung disease
IPAQ: International physical activity questionnaire
KCO: Carbon monoxide transfer coefficient
LA: Left atrial diameter
LVEDD: Left ventricular end-diastolic diameter
LVEF: Left ventricular ejection fraction
LVESD: Left ventricular end-systolic diameter
mMRC: Modified Medical Research Council
RAAS: Direct renin inhibitor and mineralocorticoid receptor antagonists,
SD: Standard deviations
TLCO: Diffusing capacity for carbon monoxide (transfer factor)
