# Supplementary material for: Cardiovascular predictors of mortality and exacerbations in patients with COPD
Source: Sci Rep. 2022 Dec 19;12:21882. doi: 10.1038/s41598-022-25938-0 (PMC9763357; doi:10.1038/s41598-022-25938-0)
Supplement: Supplementary file 1 — Supplementary Information. [file 41598_2022_25938_MOESM1_ESM.docx]

**Cardiovascular predictors of mortality and exacerbations in patients with COPD**

Peter Alter^1^, Tanja Lucke^2^, Henrik Watz^3^, Stefan Andreas^4^, Kathrin Kahnert^5^,
Franziska C. Trudzinski^6^, Tim Speicher^1^, Sandra Söhler^1^, Robert Bals^7^, Benjamin Waschki^8^, Tobias Welte^9^, Klaus F. Rabe^10^, Jørgen Vestbo^11^, Emiel F. M. Wouters^12^,
Claus F. Vogelmeier^1^, Rudolf A. Jörres^2^

# Additional file 1

# Supplemental Results

**Supplementary Figure 1. (A) Survival as function of coronary artery disease without reported infarction and (B) as function of hypertension.**


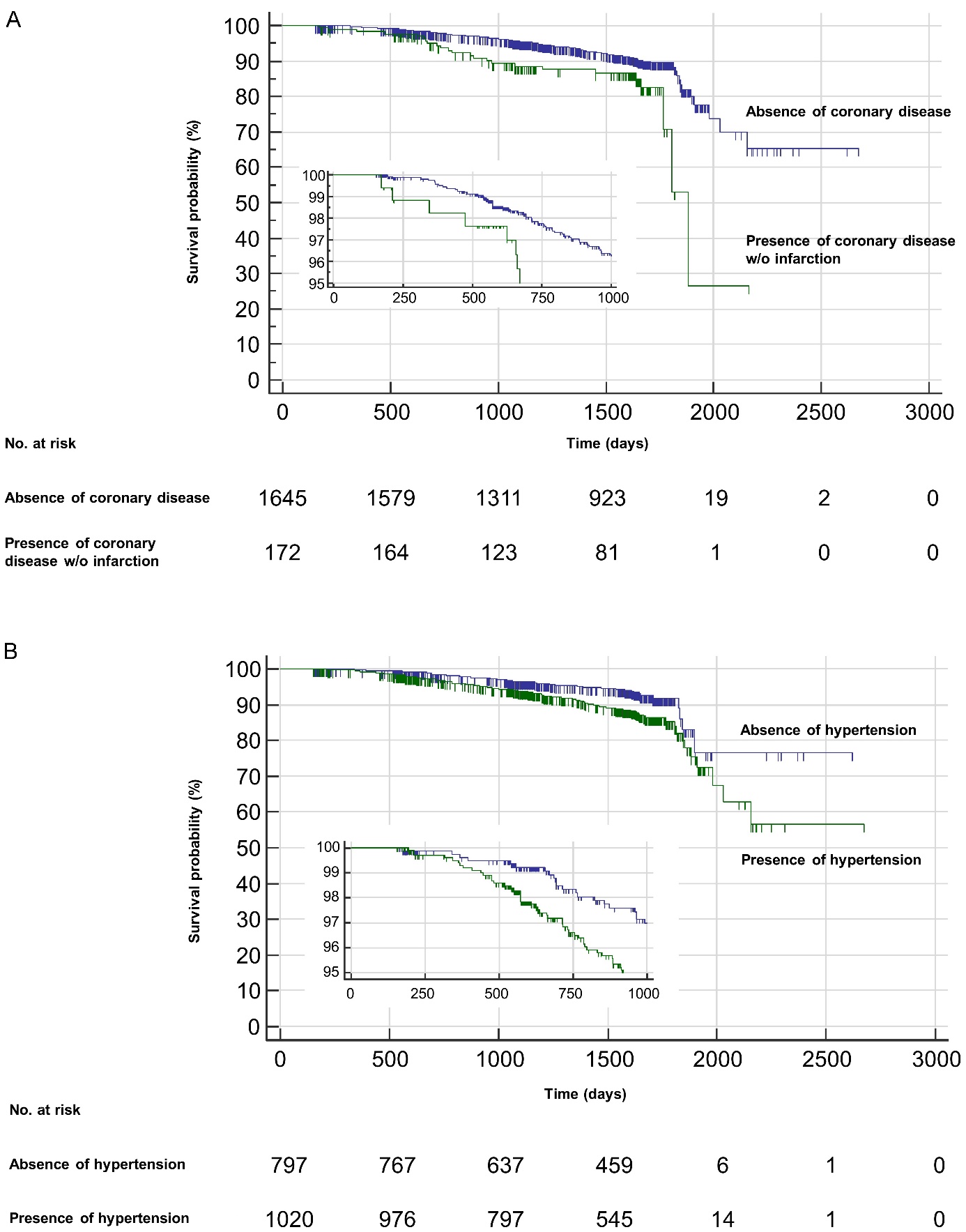


(A) Kaplan-Meier estimates of survival for the two groups of patients with COPD who either had coronary artery disease without myocardial infarction, or no coronary artery disease. (B) Kaplan-Meier estimates of survival for the two groups of patients with COPD who either had hypertension or not.

**Supplementary Table 1. Characteristics of survivors and deceased patients.**

| Variable | Survivors  n = 1664 | Deceased  n = 153 | p value |
| --- | --- | --- | --- |
| Sex (male/female) | 1003 (60.3%) / 661 (39.7%) | 115 (75.2%) /  38 (24.8%) | <0.001 |
| Age (y) | 64.4 ± 8.3 | 69 ± 7.6 | <0.001 |
| BMI (kg/m^2^) | 26.8 ± 5.1 | 26.7 ± 5.3 | 0.736 |
| Current smoking | 400 (24.0%) | 41 (26.8%) | 0.432 |
| FEV_1_ at baseline (% predicted GLI) | 54.9 ± 18.0 | 48.4 ± 18.4 | <0.001 |
| TLCO at baseline (% predicted GLI) | 57.7 ± 21.2 | 48.4 ± 19.5 | <0.001 |
| Delta/y FEV_1_ (% predicted GLI) | -1.40 ± 5.36 | -2.32 ± 6.46 | 0.047 |
| Delta/y TLCO (% predicted GLI) | -2.40 ± 7.36 | -4.60 ± 9.30 | <0.001 |
| Coronary artery disease w/o infarction | 148 (8.9%) | 24 (15.7%) | 0.009 |
| Heart failure | 77 (4.6%) | 11 (7.2%) | 0.166 |
| Rhythm disorders | 143 (8.6%) | 13 (8.5%) | 1.000 |
| Hypertension | 913 (54.9%) | 107 (69.9%) | <0.001 |
| Asthma | 306 (18.4%) | 28 (18.3%) | 1.000 |
| Sleep apnoea | 174 (10.5%) | 27 (17.6%) | 0.010 |
| Diabetes | 206 (12.4%) | 30 (19.6%) | 0.016 |
| Hyperlipidaemia | 732 (44.0%) | 72 (47.1%) | 0.497 |
| Hyperuricaemia | 290 (17.4%) | 44 (28.8%) | <0.001 |
| Osteoporosis | 252 (15.1%) | 29 (19.0%) | 0.242 |
| Psychological disorders | 396 (23.8%) | 41 (26.8%) | 0.429 |
| IPAQ (score points) | 4614 ± 4929 | 3133 ± 3743 | <0.001 |
| High exacerbation risk at baseline | 551 (33.1%) | 65 (42.5%) | 0.020 |
| Increased symptoms at baseline | 729 (43.8%) | 90 (58.8%) | <0.001 |
| Antiplatelet agents | 410 (24.6%) | 40 (26.1%) | 0.696 |
| Beta-blockers | 359 (21.6%) | 52 (34.0%) | <0.001 |
| RAAS inhibitors | 716 (43.0%) | 70 (45.8%) | 0.551 |
| Diuretics | 468 (28.1%) | 63 (41.2%) | 0.001 |
| Lipid-lowering drugs | 397 (23.9%) | 38 (24.8%) | 0.767 |

BMI = body-mass index; FEV_1_ = forced expiratory volume in 1 second; TLCO = diffusing capacity for carbon monoxide; Delta/y = annual decline from baseline expressed as %predicted at baseline; IPAQ = International Physical Activity Questionnaire; RAAS = Renin-angiotensin-aldosterone system. Increased symptoms correspond to Global Initiative for Chronic Obstructive Lung Disease (GOLD) groups B or D and increased exacerbation risk to GOLD groups C or D. Comparisons between groups were performed by chi-square statistics and unpaired t-tests. All predictors except Delta/y refer to baseline values.

**Supplementary Table 2. Results of Cox regression analysis to predict mortality.**

| **Predictor of mortality** | **Hazard ratio  (95% CI)** | **p value** |
| --- | --- | --- |
| Sex (male) | 1.648 (1.091, 2.489) | 0.018 |
| Age (per 5 years) | 1.531 (1.351, 1.734) | <0.001 |
| BMI (kg/m^2^) | 0.997 (0.958, 1.038) | 0.890 |
| Current smoking | 1.742 (1.182, 2.569) | 0.005 |
| FEV_1_ at baseline (% predicted GLI, per 5%) | 0.924 (0.864, 0.988) | 0.020 |
| TLCO at baseline (% predicted GLI, per 5%) | 0.896 (0.845, 0.950) | <0.001 |
| Delta/y FEV_1_ (% predicted GLI, per 5%) | 1.159 (0.954, 1.409) | 0.138 |
| Delta/y TLCO (% predicted GLI, per 5%) | 1.238 (1.086, 1.412) | 0.001 |
| Coronary artery disease w/o infarction | 1.967 (1.223, 3.163) | 0.005 |
| Heart failure | 1.132 (0.553, 2.316) | 0.735 |
| Rhythm disorders | 0.674 (0.344, 1.326) | 0.252 |
| Hypertension | 1.517 (1.011, 2.276) | 0.044 |
| Asthma | 1.110 (0.720, 1.713) | 0.637 |
| Sleep apnoea | 1.583 (0.986, 2.542) | 0.057 |
| Diabetes | 1.191 (0.744, 1.907) | 0.467 |
| Hyperlipidaemia | 0.876 (0.594, 1.292) | 0.504 |
| Hyperuricaemia | 1.391 (0.941, 2.057) | 0.098 |
| Osteoporosis | 1.174 (0.755, 1.826) | 0.476 |
| Psychological disorders | 1.542 (1.048, 2.268) | 0.028 |
| IPAQ (per 1000 score points) | 0.945 (0.903, 0.989) | 0.013 |
| High exacerbation risk at baseline | 1.200 (0.852, 1.691) | 0.297 |
| Increased symptoms at baseline | 0.940 (0.636, 1.389) | 0.755 |
| Antiplatelet agents | 0.705 (0.461, 1.079) | 0.107 |
| Beta-blockers | 1.564 (1.072, 2.281) | 0.020 |
| RAAS inhibitors | 0.770 (0.522, 1.136) | 0.188 |
| Diuretics | 1.097 (0.746, 1.613) | 0.639 |
| Lipid-lowering drugs | 0.953 (0.584, 1.554) | 0.847 |

BMI = body-mass index; FEV_1_ = forced expiratory volume in 1 second; TLCO = diffusing capacity for carbon monoxide; Delta/y = annual decline from baseline expressed as %predicted at baseline; IPAQ = International Physical Activity Questionnaire; mMRC = modified medical research scale; RAAS = Renin-angiotensin-aldosterone system. Increased symptoms correspond to Global Initiative for Chronic Obstructive Lung Disease (GOLD) groups B or D and increased exacerbation risk to GOLD groups C or D. All predictors except Delta/y refer to baseline values.

## Predictors of mortality

When applying stepwise forward selection to account for potential missing of statistical significance due to collinearities, the major result was confirmed but sleep apnoea and hyperuricaemia were identified as additional predictors (p<0.05 each) after the reduction of the number of predictors. The same predictors were identified as statistically significant also by backward selection.

## Sensitivity analyses

***Coronary artery disease***

Only the presence of coronary artery disease without myocardial infarction was a robust, significant predictor of mortality. When replacing this variable by coronary artery disease (without specifying myocardial infarction status), this variable was never significant (p>0.10). Regarding annual exacerbation rate, coronary artery disease without myocardial infarction was not significant (see Supplementary Table 4). After replacing this variable by coronary artery disease without specification, this variable was still not significant (p>0.10).

When excluding patients with coronary artery disease without specification, the results for mortality remained the same regarding age, smoking status, baseline FEV_1_, TLCO % predicted and the annual fall in TLCO % predicted, IPAQ and psychological disorders. There was, however, no longer a significant association with beta-blockers, and there were still no significant associations with other cardiac disorders. The number of patients with coronary artery disease was too small (n=330) to allow for analyses comparable in statistical power to those of the group without coronary artery disease (n=1487).

***Respiratory medication***

Respiratory medication is known to be relevant for COPD outcomes. When repeating the mortality analyses with additional predictors indicating the presence of any LABA, or any LAMA, or any ICS, none of these were statistically significant. The same was true when including LABA+ICS, LABA+LAMA, or triple therapy as predictors, while all predictors identified without respiratory therapy remained significant. When repeating the analogous computation for the mean annual exacerbation rate, LABA, ICS and triple therapy were significantly related to a reduction in exacerbation rate, while the predictors identified without respiratory medication remained significant and unaffected by this extension.

**Supplementary Table 3. Characteristics of patients with low and high mean annual exacerbation rate.**

| Variable | Low exacerbation history category  n = 1239 | High exacerbation history category  n = 578 | p value |
| --- | --- | --- | --- |
| Sex (male/female) | 776 (62.6%) / 463 (37.4%) | 342 (59.2%) / 236 (40.8%) | 0.162 |
| Age (y) | 65.1 ± 8.4 | 64.3 ± 8.1 | 0.067 |
| BMI (kg/m^2^) | 26.7 ± 5 | 26.9 ± 5.4 | 0.486 |
| Current smoking | 335 (27.0%) | 106 (18.3%) | <0.001 |
| FEV_1_ at baseline (% predicted GLI) | 57.8 ± 17.9 | 46.7 ± 16.2 | <0.001 |
| TLCO at baseline (% predicted GLI) | 60 ± 21.3 | 50.4 ± 19.6 | <0.001 |
| Delta/y FEV_1_ (% predicted GLI) | -1.4 ± 4.9 | -1.6 ± 6.5 | 0.626 |
| Delta/y TLCO (% predicted GLI) | -2.3 ± 6.8 | -3.1 ± 8.9 | 0.033 |
| Coronary artery disease w/o infarction | 100 (8.1%) | 72 (12.5%) | 0.003 |
| Heart failure | 51 (4.1%) | 37 (6.4%) | 0.045 |
| Rhythm disorders | 83 (6.7%) | 73 (12.6%) | <0.001 |
| Hypertension | 678 (54.7%) | 342 (59.2%) | 0.076 |
| Asthma | 190 (15.3%) | 144 (24.9%) | <0.001 |
| Sleep apnoea | 115 (9.3%) | 86 (14.9%) | <0.001 |
| Diabetes | 138 (11.1%) | 98 (17.0%) | <0.001 |
| Hyperlipidaemia | 535 (43.2%) | 269 (46.5%) | 0.187 |
| Hyperuricaemia | 223 (18.0%) | 111 (19.2%) | 0.559 |
| Osteoporosis | 159 (12.8%) | 122 (21.1%) | <0.001 |
| Psychological disorders | 265 (21.4%) | 172 (29.8%) | <0.001 |
| IPAQ (score points) | 4914.5 ± 5045.4 | 3581.6 ± 4292.5 | <0.001 |
| High exacerbation risk at baseline | 210 (16.9%) | 406 (70.2%) | <0.001 |
| Increased symptoms at baseline | 446 (36.0%) | 373 (64.5%) | <0.001 |
| Antiplatelet agents | 296 (23.9%) | 154 (26.6%) | 0.220 |
| Beta-blockers | 270 (21.8%) | 141 (24.4%) | 0.229 |
| RAAS inhibitors | 507 (40.9%) | 279 (48.3%) | 0.004 |
| Diuretics | 322 (26.0%) | 209 (36.2%) | <0.001 |
| Lipid-lowering drugs | 300 (24.2%) | 135 (23.4%) | 0.723 |

BMI = body-mass index; FEV_1_ = forced expiratory volume in 1 second; TLCO = diffusing capacity for carbon monoxide; Delta/y = annual decline from baseline expressed as % predicted at baseline; IPAQ = International Physical Activity Questionnaire; RAAS = Renin-angiotensin-aldosterone system. Increased symptoms correspond to Global Initiative for Chronic Obstructive Lung Disease (GOLD) groups B or D and high exacerbation risk to GOLD groups C or D at baseline. Comparisons between groups were performed by chi-square statistics and unpaired t-tests. All predictors except Delta/y refer to baseline values.

**Supplementary Table 4. Results of Poisson regression analysis to predict the mean number of exacerbations per year**.

| **Predictor of increased exacerbation occurrence** | **Odds ratio  (95 % CI)** | **p value** |
| --- | --- | --- |
| Sex (male) | 0.930 (0.843, 1.027) | 0.152 |
| Age (per 5 years) | 0.995 (0.966, 1.025) | 0.726 |
| BMI (kg/m^2^) | 0.995 (0.985, 1.005) | 0.328 |
| Current smoking | 0.922 (0.822, 1.034) | 0.164 |
| FEV_1_ at baseline (% predicted GLI, per 5%) | 0.952 (0.936, 0.969) | <0.001 |
| TLCO at baseline (% predicted GLI, per 5%) | 0.995 (0.981, 1.010) | 0.509 |
| Delta/y FEV_1_ (% predicted GLI, per 5%) | 1.052 (1.008, 1.097) | 0.019 |
| Delta/y TLCO (% predicted GLI, per 5%) | 1.011 (0.981, 1.042) | 0.482 |
| Coronary artery disease w/o infarction | 1.033 (0.888, 1.203) | 0.672 |
| Heart failure | 0.999 (0.819, 1.220) | 0.993 |
| Rhythm disorders | 1.128 (0.965, 1.317) | 0.130 |
| Hypertension | 0.988 (0.884, 1.104) | 0.833 |
| Asthma | 1.075 (0.961, 1.201) | 0.207 |
| Sleep apnoea | 1.086 (0.943, 1.250) | 0.252 |
| Diabetes | 1.120 (0.979, 1.282) | 0.098 |
| Hyperlipidaemia | 1.017 (0.915, 1.131) | 0.754 |
| Hyperuricaemia | 1.019 (0.905, 1.147) | 0.758 |
| Osteoporosis | 1.036 (0.920, 1.167) | 0.561 |
| Psychological disorders | 1.098 (0.991, 1.217) | 0.075 |
| IPAQ (per 1000 score points) | 0.992 (0.982, 1.002) | 0.105 |
| High exacerbation risk at baseline | 2.204 (2.005, 2.422) | <0.001 |
| Increased symptoms at baseline | 1.176 (1.058, 1.306) | 0.003 |
| Antiplatelet agents | 0.959 (0.851, 1.082) | 0.499 |
| Beta-blockers | 1.006 (0.898, 1.128) | 0.918 |
| RAAS inhibitors | 1.072 (0.956, 1.201) | 0.235 |
| Diuretics | 1.038 (0.930, 1.158) | 0.508 |
| Lipid-lowering drugs | 0.969 (0.845, 1.111) | 0.655 |

BMI = body-mass index; FEV_1_ = forced expiratory volume in 1 second; TLCO = diffusing capacity for carbon monoxide; Delta/y = annual decline from baseline expressed as % predicted at baseline; IPAQ = International Physical Activity Questionnaire; mMRC = modified medical research scale; RAAS = Renin-angiotensin-aldosterone system. Increased symptoms correspond to Global Initiative for Chronic Obstructive Lung Disease (GOLD) groups B or D and high exacerbation risk to GOLD groups C or D at baseline. All predictors except Delta/y refer to baseline values.

# Supplemental Discussion

Echocardiographic measures were nearly always in the normal clinical range ^1,2^. As a consequence, the variations within this range were probably not strong enough to show an effect on mortality and annual exacerbation rate when included as additional predictors. Irrespective of this, there may be biomarkers in COPD patients for which variations even within ranges commonly considered as normal can be of importance. For example, in previous analyses of COSYCONET data, a slight increase in the level of high-sensitivity troponin or small alterations in the concentration of oxygenated haemoglobin were linked to mortality ^3,4^. Similarly, a reduction in left ventricular size, although remaining within the normal range, independently contributed to dyspnoea in patients with COPD ^5^.

It is noteworthy that the presence of cardiovascular comorbidities at baseline did not contribute to the mean rate of exacerbations. Other studies found that chronic bronchial infection, a potential trigger of exacerbations, was associated with an increased incidence of acute cardiovascular events. Probably these events represent a different type of cardiovascular comorbidity compared to those included in our analyses. Our findings underscore the need of a cautious definition of cardiovascular comorbidities in COPD ^6^.

Several studies have shown that reduced exercise capacity and physical activity ^7,8^ are linked to mortality in COPD. Our observation of a relationship to physical activity, as quantified by IPAQ, was in line with this. We preferred this instrument over other measures such as the 6-minute walking distance (6-MWD), as there are many locations in which the 6-MWD cannot be easily determined, while the IPAQ can. When tentatively replacing IPAQ by 6-MWD in the analyses, the significant predictors of mortality, particularly baseline diffusing capacity and its decline, the presence of coronary artery disease without prior infarction, and beta-blockers remained the same, while 6-MWD showed a similar, significant association as IPAQ. When repeating the analysis for the mean annual exacerbation rate, the significant relationship to baseline FEV_1_ and its decline was also unaffected, as well as the relationship to baseline symptoms and exacerbation risk; conversely, 6-MWD was not significant, as had been found for IPAQ.

Drop-out of patients during follow-up meant that only 836 patients completed visit 5, none of whom died during the subsequent observational period. When excluding the patients who completed visit 5, the decline in TLCO and the presence of coronary artery disease without remote infarction were still significant predictors of mortality. The same was true when patients with last visit 2 were included or excluded in order to account for the fact that visit 2 occurred only 6 months after visit 1 and thus the changes in lung function were based on a short time period. We therefore consider it unlikely that differential loss of patients played a relevant role regarding our main conclusions.

# References

1 Alter, P. *et al.* Left ventricular volume and wall stress are linked to lung function impairment in COPD. *Int J Cardiol* **261**, 172-178 (2018). <https://doi.org:10.1016/j.ijcard.2018.02.074>

2 Alter, P. *et al.* Airway obstruction and lung hyperinflation in COPD are linked to an impaired left ventricular diastolic filling. *Respir Med* **137**, 14-22 (2018). <https://doi.org:10.1016/j.rmed.2018.02.011>

3 Waschki, B. *et al.* High-sensitivity troponin I and all-cause mortality in patients with stable COPD: an analysis of the COSYCONET study. *Eur Respir J* **55** (2020). <https://doi.org:10.1183/13993003.01314-2019>

4 Trudzinski, F. C. *et al.* Associations of oxygenated hemoglobin with disease burden and prognosis in stable COPD: Results from COSYCONET. *Sci Rep* **10**, 10544 (2020). <https://doi.org:10.1038/s41598-020-67197-x>

5 Alter, P. *et al.* Prevalence of cardiac comorbidities, and their underdetection and contribution to exertional symptoms in COPD: results from the COSYCONET cohort. *Int J Chron Obstruct Pulmon Dis* **14**, 2163-2172 (2019). <https://doi.org:10.2147/COPD.S209343>

6 Martinez-Garcia, M. A. *et al.* Chronic bronchial infection and incident cardiovascular events in chronic obstructive pulmonary disease patients: A long-term observational study. *Respirology* **26**, 776-785 (2021). <https://doi.org:10.1111/resp.14086>

7 Celli, B. R. *et al.* The body-mass index, airflow obstruction, dyspnea, and exercise capacity index in chronic obstructive pulmonary disease. *N Engl J Med* **350**, 1005-1012 (2004). <https://doi.org:10.1056/NEJMoa021322>

8 Waschki, B. *et al.* Physical activity is the strongest predictor of all-cause mortality in patients with COPD: a prospective cohort study. *Chest* **140**, 331-342 (2011). <https://doi.org:10.1378/chest.10-2521>
